# Association Between Cervical Drainage and Early Post-Thyroidectomy Outcomes: A Systematic Review and Meta-Analysis

**DOI:** 10.3390/jcm15072494

**Published:** 2026-03-24

**Authors:** Michael Kostares, Evangelos Kostares, Maria Kakazani, Marina Karaiskou, Paul Stampouloglou, Maria Kantzanou, Spiridon Laskaris, Maria Piagkou

**Affiliations:** 1 Department of Anatomy, National and Kapodistrian University of Athens, 106 79 Athens, Greece; mapian@med.uoa.gr; 2 Department of Otorhinolaryngology—Head and Neck Surgery, “Metaxa” Memorial Anticancer Hospital, 185 37 Piraeus, Greece; 3 Department of Oral and Maxillofacial Surgery, National and Kapodistrian University of Athens, “Evangelismos” General Hospital, 106 76 Athens, Greece; kostevang@med.uoa.gr; 4 Department of Microbiology, Medical School, National and Kapodistrian University of Athens, 115 27 Athens, Greece; mkatzan@med.uoa.gr

**Keywords:** thyroidectomy, cervical drainage, postoperative complications, surgical site infection, hematoma, length of hospital stay, meta-analysis

## Abstract

**Background/Objectives**: Cervical drainage has traditionally been used after thyroidectomy to reduce postoperative fluid accumulation and mitigate bleeding-related complications. However, advances in surgical technique, perioperative hemostasis, and postoperative care pathways have led to an increase in the use of short-stay and outpatient thyroidectomy, prompting renewed evaluation of the role of routine drainage. The objective of this systematic review and meta-analysis was to examine the association between postoperative cervical drainage and postoperative outcomes following thyroidectomy. **Methods**: A systematic literature search was conducted across PubMed/MEDLINE, Google Scholar, Semantic Scholar, and the Cochrane Central Register of Controlled Trials to identify studies comparing thyroidectomy with versus without cervical drainage. Studies published between January 2005 and January 2026 were eligible for inclusion. Randomized controlled trials and non-randomized comparative studies involving adult patients were included. The outcomes of interest were cervical hematoma, surgical site infection (SSI), seroma formation, postoperative bleeding, reoperation, and length of hospital stay. Random-effects meta-analyses were performed using odds ratios for binary outcomes and mean differences for continuous outcomes. Sensitivity and influence analyses were conducted to assess robustness. The results were additionally examined in prespecified sensitivity analyses restricted to randomized trials, and study-design-stratified estimates are presented. **Results**: Thirty studies comprising 2810 patients were included. Drain use was not statistically significantly associated with postoperative cervical hematoma (OR 1.28, 95% CI 0.93–1.75; *p* = 0.124). In contrast, drain use was associated with a significantly increased risk of surgical site infection (OR 2.04, 95% CI 1.46–2.85; *p* = 0.0002) and a significantly longer postoperative length of hospital stay (mean difference 1.96 days, 95% CI 0.42–3.50; *p* = 0.016). No statistically significant associations were observed between drainage and seroma formation (OR 0.95, 95% CI 0.70–1.30; *p* = 0.750), postoperative bleeding (OR 1.26, 95% CI 0.85–1.86; *p* = 0.228), or reoperation (OR 0.89, 95% CI 0.59–1.32; *p* = 0.525). Sensitivity and influence analyses demonstrated consistent results across analytical approaches and study designs. **Conclusions**: In thyroidectomy, routine cervical drainage is not associated with a reduction in bleeding-related complications and is associated with adverse recovery-related outcomes, including increased risk of surgical site infection and prolonged hospitalization. Overall, the findings indicate that routine cervical drainage after thyroidectomy offers no clear advantage in preventing postoperative complications and may be associated with adverse postoperative outcomes. Routine cervical drainage after thyroidectomy was not associated with a protective effect on complications and showed associations with less favorable recovery-related outcomes.

## 1. Introduction

Thyroidectomy is the most frequently performed endocrine surgical procedure worldwide, and its perioperative management continues to evolve as surgical techniques, hemostatic technologies, and postoperative care pathways increasingly favor early mobilization and shorter length of hospital stay [1,2,3]. Historically, the placement of a cervical drain has been incorporated into postoperative practice in many surgical units, with the intention of reducing dead space and facilitating the evacuation of blood or lymphatic fluid, thereby aiming to limit postoperative fluid accumulation and wound-related complications [4,5]. Although this rationale has been widely accepted, the clinical effectiveness of routine drainage in preventing adverse postoperative events has been increasingly questioned. Postoperative cervical hematoma, although uncommon, represents the most serious early complication following thyroidectomy because of its potential to cause rapid airway compromise and the need for urgent intervention [6]. In addition to hematoma, other early postoperative concerns—including seroma formation, surgical site infection, postoperative pain, and prolonged hospitalization—remain clinically relevant, as they directly influence patient recovery, healthcare utilization, and the feasibility of short-stay or outpatient thyroidectomy in appropriately selected patients [1,7].

The role of postoperative cervical drainage in thyroidectomy remains an area of clinical consideration. Drain placement has traditionally been used with the aim of reducing postoperative fluid accumulation and facilitating the early detection of bleeding. At the same time, ongoing developments in surgical technique, intraoperative hemostasis, and postoperative care pathways have influenced routine perioperative practices. Despite the widespread performance of thyroidectomy and the continued use of drains in selected settings, the overall impact of drainage on early postoperative outcomes remains incompletely defined. These considerations support the need for a structured synthesis of the available comparative evidence.

The objective of the present systematic review and meta-analysis was to examine the association between postoperative cervical drainage and early postoperative outcomes following thyroidectomy, including cervical hematoma, seroma formation, surgical site infection, postoperative bleeding, reoperation, and length of hospital stay.

## 2. Materials and Methods

### 2.1. Study Design and Reporting Standards

This study was conducted as a systematic review and meta-analysis comparing the use of postoperative cervical drains versus no drainage following thyroidectomy. The methodology was defined a priori and aimed to ensure rigorous extraction and synthesis of postoperative complication data across heterogeneous study designs and clinical settings. The review process and reporting adhered to the Cochrane Handbook for Systematic Reviews of Interventions and to the Preferred Reporting Items for Systematic Reviews and Meta-Analyses (PRISMA) guidelines [8]. The PRISMA checklist is available in the Supplementary Materials (Table S1). This review was registered in the International Prospective Register of Systematic Reviews (PROSPERO) under the registration number CRD420261304442.

### 2.2. Literature Search Strategy

A comprehensive literature search was performed to identify studies evaluating outcomes following thyroidectomy with or without the use of surgical drains. Electronic databases systematically searched included PubMed/MEDLINE, Google Scholar, Semantic Scholar, and the Cochrane Central Register of Controlled Trials. The search strategy combined Medical Subject Headings (MeSH) and free-text terms related to thyroidectomy, postoperative drainage, neck drains, and postoperative complications. Free-text terms were searched within titles and abstracts and included keywords related to thyroidectomy (“thyroid”), drainage (“drain*”), and the perioperative period (“post*”, “peri*”, “intra*”, “operat*”, “surg*”). The complete search strategy for each database is provided in the Supplementary Materials (Table S2).

### 2.3. Eligibility Criteria

Studies were considered eligible if they met the following criteria: (i) inclusion of adult patients undergoing thyroidectomy (total, near-total, or hemithyroidectomy/lobectomy), (ii) a direct comparison between a drain group and a no-drain group, and (iii) reporting of at least one postoperative outcome of interest. Both randomized controlled trials and non-randomized comparative studies were eligible for inclusion, and, to ensure clinical relevance and methodological comparability, eligibility was restricted to studies published from January 2005 to January 2026. Non-randomized comparative studies included retrospective cohort studies, retrospective case–control studies, and prospective comparative cohort designs, with a clearly defined drain versus no-drain comparison.

Non-English publications, studies employing more than two intervention arms (unless they provided a clearly defined and directly extractable comparison between a drain and a no-drain group), case reports, case series without a comparative group, editorials, letters, and conference abstracts, and studies involving pediatric populations were excluded. Finally, studies were excluded if postoperative outcomes of interest were not explicitly reported or could not be reliably extracted from the published data.

### 2.4. Study Selection and Data Extraction

All records identified through the search strategy were screened independently at the title and abstract level. Potentially relevant studies underwent full-text assessment for eligibility. Discrepancies at any stage of study selection were resolved through discussion and consensus. When necessary, a third reviewer was consulted to resolve disagreements.

Data extraction was performed using a standardized extraction form. Extracted variables included first author, year of publication, country, study design, total sample size, number of patients in the drain and no-drain groups, and postoperative outcomes. A strict double-verification rule was applied during data extraction. Variables were recorded only when explicitly reported and independently identifiable in at least two sections of the manuscript. When a variable or outcome was not explicitly stated, it was recorded as missing rather than assumed. No data were inferred or imputed based on clinical reasoning alone. For binary outcomes, the number of events and total number of patients in each treatment arm were extracted. Zero-event values were recorded only when the original authors explicitly stated the absence of events in each group. Outcomes that were not reported were treated as missing data. When reported, the criterion used for drain removal was extracted and standardized to milliliters per 24 h. This variable was retained for descriptive reporting and exploratory analyses but was not used as a primary eligibility criterion.

### 2.5. Outcomes of Interest

The postoperative outcomes of interest included hematoma, surgical site infection (SSI), seroma formation, postoperative bleeding, reoperation, and length of hospital stay. These outcomes were selected based on their clinical relevance and consistency with prior literature evaluating the impact of postoperative drainage in thyroidectomy. Hematoma, SSI, and seroma were classified as local postoperative complications and were extracted as distinct binary outcomes. Hematoma was recorded only when explicitly described as a wound or cervical hematoma. Seroma was recorded only when clearly reported as a postoperative fluid collection requiring clinical recognition or intervention. Surgical site infection was recorded according to the original authors’ definitions. Postoperative bleeding was treated as a separate outcome and included postoperative bleeding or hemorrhage as explicitly reported by the study authors. Bleeding events were not equated with hematoma unless the original study explicitly stated that the terms referred to the same clinical event. Reoperation was defined as return to the operating theatre, surgical re-exploration, or reoperation as explicitly described by the authors, regardless of the underlying indication. Length of hospital stay was analyzed as a continuous outcome and extracted as group-specific means and standard deviations when reported. Studies reporting length of stay using medians or ranges without convertible summary statistics were not included in the pooled analysis of this outcome. Composite morbidity outcomes were not calculated unless explicitly reported as patient-level composite endpoints in the original studies. Event counts were not summed across complications to avoid double-counting in the presence of potential overlap between outcomes.

### 2.6. Risk of Bias Assessment

Given the inclusion of both randomized and non-randomized studies, risk of bias was assessed according to study design. Randomized controlled trials were evaluated using the Cochrane Risk of Bias tool [9], whereas observational studies were assessed using the Newcastle–Ottawa Scale [10]. Risk-of-bias assessments were performed to inform study interpretation and sensitivity analyses; however, detailed study-level assessments were not incorporated into the main text and are available in Table S3.

### 2.7. Statistical Analysis

All statistical analyses were performed using R statistical software (R Foundation for Statistical Computing, Vienna, Austria) with RStudio Version 2026.01.0+392 (Posit Software, PBC, Boston, MA, USA), using validated packages for meta-analysis and generalized linear mixed modeling [11]. For dichotomous outcomes, pooled effect estimates were calculated as odds ratios (ORs) with corresponding 95% confidence intervals (CIs) [12]. Random-effects models were employed to account for both within-study and between-study variability. Between-study variance (τ^2^) was estimated using restricted maximum likelihood (REML) [13,14]. To improve the reliability of inference, particularly in analyses with sparse event data or a limited number of studies, Hartung–Knapp adjustment was applied to random-effects model confidence intervals [15,16]. This approach was selected a priori due to its superior performance under conditions of low event rates compared with conventional DerSimonian–Laird methods. Studies reporting zero events in one treatment arm were retained using a continuity correction of 0.5 [17,18]. Studies with zero events in both treatment arms were included in the primary analysis to avoid bias associated with the systematic exclusion of clinically informative comparisons [18]. The influence of double-zero studies was explored through prespecified sensitivity analyses. Statistical heterogeneity was assessed using Cochran’s Q statistic and quantified using the I^2^ statistic, with τ and τ^2^ reported as absolute measures of between-study variability. Studies were included on an outcome-specific basis; a study could contribute data to one outcome analysis while being excluded from others due to missing or non-extractable information. Sensitivity analyses were performed to assess the robustness of the primary findings. These included the exclusion of studies with zero events in both treatment arms, alternative effect estimation using the Peto odds ratio method, and analyses restricted to randomized controlled trials only. Sensitivity analyses were interpreted based on the consistency of effect direction and magnitude rather than statistical significance alone. Influence diagnostics were conducted using leave-one-out analyses and influence measures derived from random-effects models to identify studies exerting a disproportionate influence on pooled estimates or heterogeneity [19]. As an advanced robustness analysis, one-stage generalized linear mixed-effects models were fitted for binary outcomes, incorporating study-level clustering through random effects [20]. These models were used as complementary analyses to the conventional two-stage meta-analytic approach. Pooling across study designs was prespecified to provide an overall estimate of comparative associations across clinical settings. Study-design-stratified analyses and sensitivity analyses restricted to randomized controlled trials were conducted to assess consistency across designs and to guard against potential design-related bias. All statistical tests were two-sided, and a *p* value < 0.05 was considered statistically significant. Greater emphasis was placed on effect estimates and confidence intervals rather than on *p* values alone.

## 3. Results

### 3.1. Study Selection and Characteristics

**Figure 1:**
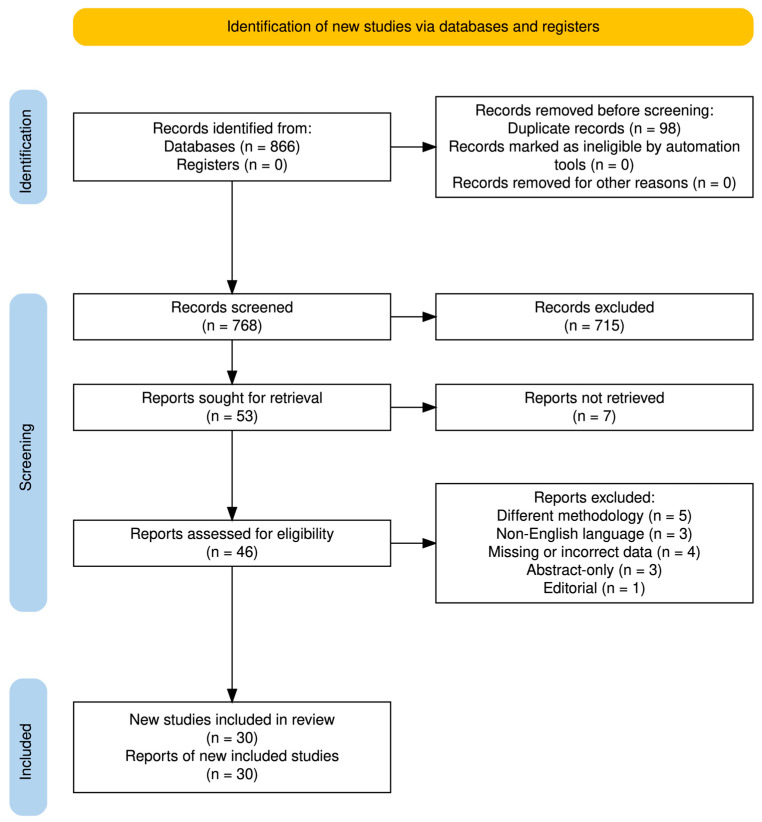
PRISMA flow diagram of the study selection process.

The study selection process is summarized in the PRISMA flow diagram (Figure 1). Following database searching, 866 records were identified. After the removal of 98 duplicate records, 768 records underwent title and abstract screening, resulting in the exclusion of 715 records. Full-text assessment was performed for 46 articles, of which 16 were excluded based on predefined eligibility criteria.

A total of 30 studies were included. The main characteristics of the included studies are presented in Table 1. Across the included studies, 2810 patients contributed data to at least one primary binary outcome, including 1481 patients managed with drains and 1329 patients managed without drains. The number of studies and patients contributing to each analysis varied according to outcome-specific data availability. A consolidated numerical summary of pooled effect estimates, corresponding confidence intervals, *p*-values, and the number of contributing studies for each postoperative outcome is provided in Table 2.

### 3.2. Postoperative Outcomes

**Figure 2:**
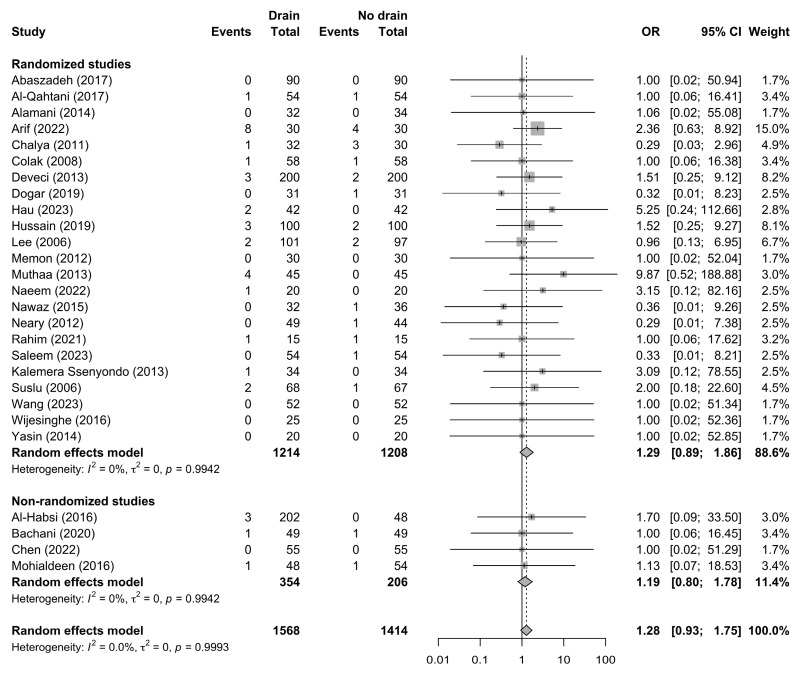
Forest plot comparing the incidence of postoperative hematoma between drain and no-drain groups. Studies are stratified according to study design. Squares represent study-specific effect estimates, with square size proportional to study weight; horizontal lines indicate 95% confidence intervals; diamonds represent pooled effect estimates with 95% confidence intervals; the vertical dotted line indicates the line of no effect [21,22,23,24,25,26,27,28,29,30,31,33,34,37,38,39,40,41,42,43,44,45,46,47,48,49,50].

Postoperative hematoma was reported in 27 studies comprising 2982 patients (1568 managed with drains and 1414 without drains), with a total of 58 hematoma events recorded. The pooled random-effects analysis demonstrated higher odds of postoperative hematoma in patients managed with drains, although the difference did not reach statistical significance (OR 1.28, 95% CI 0.93–1.75; *p* = 0.124 (Figure 2).

**Figure 3:**
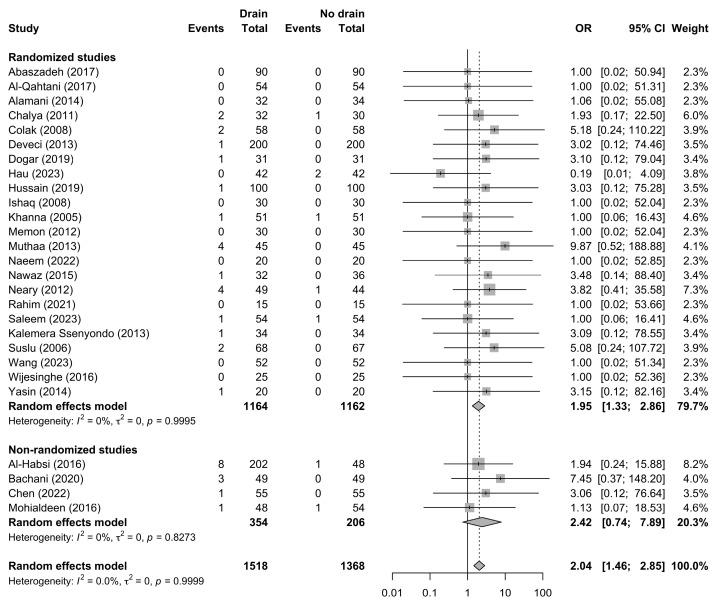
Forest plot comparing postoperative surgical site infection (SSI) rates between patients undergoing thyroidectomy with drains and those without drains, stratified by study design. Squares represent study-specific effect estimates, with square size proportional to study weight; horizontal lines indicate 95% confidence intervals; diamonds represent pooled effect estimates with 95% confidence intervals; the vertical dotted line indicates the line of no effect [21,22,23,24,26,27,28,29,30,31,33,34,35,36,38,39,40,41,42,43,44,45,46,47,48,49,50].

Surgical site infection was reported in 27 studies including 2886 patients (1518 with drains and 1368 without drains), with 43 SSI events documented. The pooled analysis showed a significantly increased risk of SSI associated with drain use compared with no-drain management (OR 2.04, 95% CI 1.46–2.85; *p* = 0.0002) (Figure 3).

**Figure 4:**
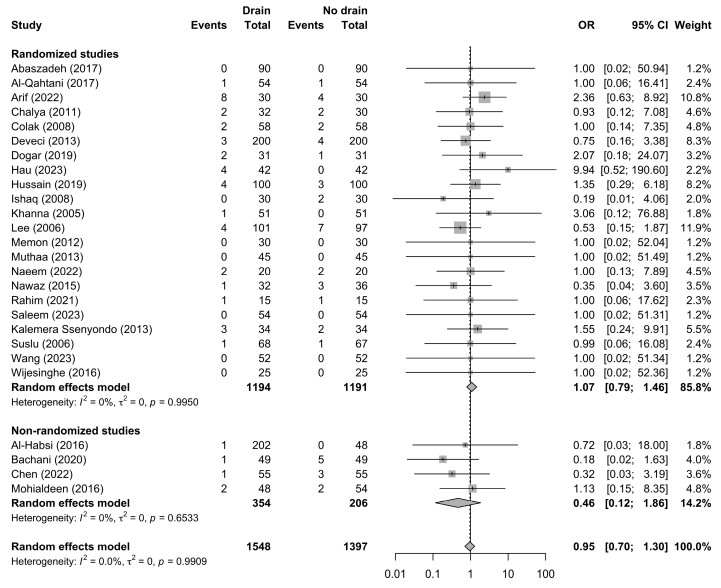
Forest plot comparing postoperative seroma formation between the drain and no-drain groups after thyroidectomy, shown overall and stratified by study design. Squares represent study-specific effect estimates, with square size proportional to study weight; horizontal lines indicate 95% confidence intervals; diamonds represent pooled effect estimates with 95% confidence intervals; the vertical dotted line indicates the line of no effect [21,22,23,25,31,32,33,34,35,36,37,38,39,40,41,42,44,45,46,47,48,49,50].

Seroma formation was evaluated in 26 studies comprising 2945 patients (1548 with drains and 1397 without drains), with a total of 89 seroma events reported. The pooled random-effects analysis did not demonstrate a significant difference in seroma risk between patients managed with drains and those without drains (OR 0.95, 95% CI 0.70–1.30; *p* = 0.750) (Figure 4).

**Figure 5:**
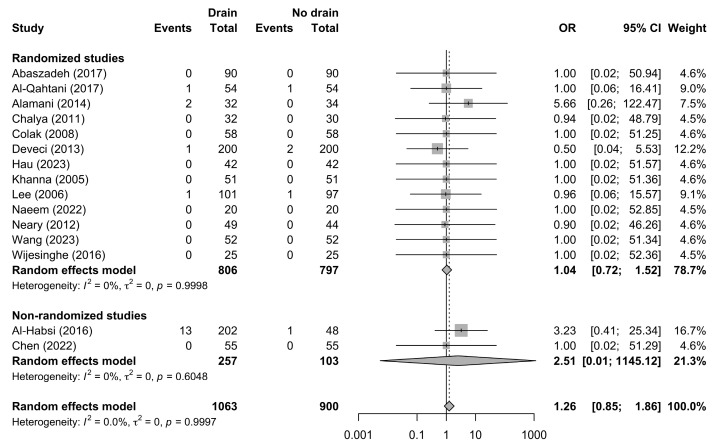
Forest plot comparing the incidence of postoperative bleeding between patients undergoing thyroidectomy with drains and those without drains, shown overall and stratified by study design. Squares represent study-specific effect estimates, with square size proportional to study weight; horizontal lines indicate 95% confidence intervals; diamonds represent pooled effect estimates with 95% confidence intervals; the vertical dotted line indicates the line of no effect [21,22,23,24,27,28,29,30,33,36,37,41,43,48,49].

Postoperative bleeding outcomes were reported in 15 studies including 1963 patients, of whom 1063 were managed with drains and 900 without drains, with a total of 23 bleeding events recorded. The pooled random-effects analysis (Mantel–Haenszel method with Hartung–Knapp adjustment) did not demonstrate a statistically significant difference in the odds of postoperative bleeding between patients managed with drains and those without drains (OR 1.26, 95% CI 0.85–1.86; *p* = 0.228) (Figure 5).

**Figure 6:**
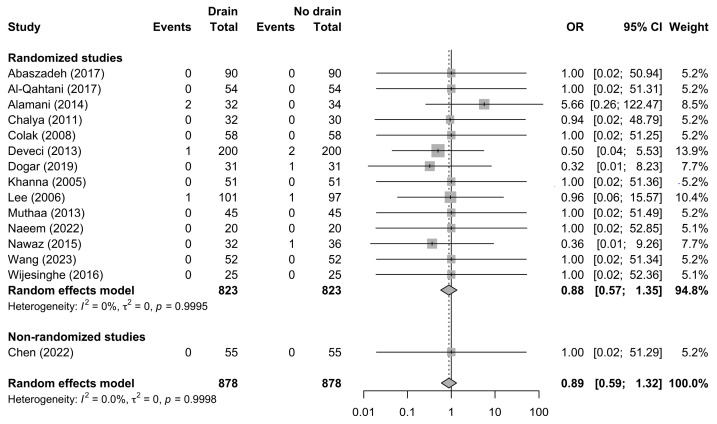
Forest plot comparing reoperation rates between the drain and no-drain groups after thyroidectomy, shown overall and stratified by study design. Squares represent study-specific effect estimates, with square size proportional to study weight; horizontal lines indicate 95% confidence intervals; diamonds represent pooled effect estimates with 95% confidence intervals; the vertical dotted line indicates the line of no effect [21,23,24,27,28,29,30,31,36,37,40,41,42,48,49].

Reoperation for postoperative complications was reported in 15 studies comprising 1756 patients, with 878 patients in each treatment group and a total of nine reoperation events. The pooled random-effects analysis did not identify a statistically significant difference in reoperation rates between patients managed with drains and those managed without drains (OR 0.89, 95% CI 0.59–1.32; *p* = 0.525) (Figure 6).

**Figure 7:**
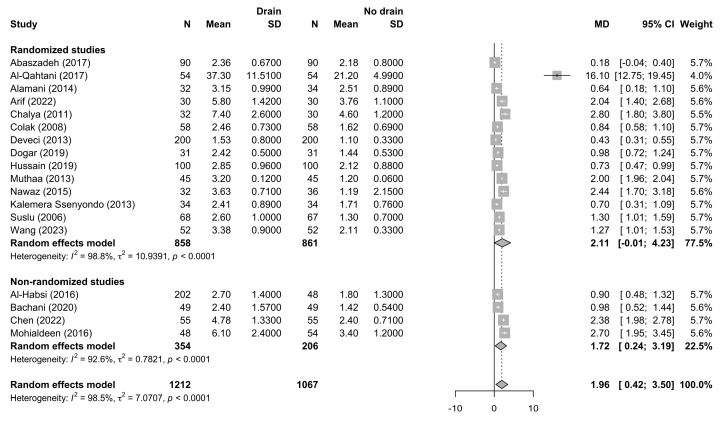
Forest plot comparing postoperative length of hospital stay (LOS) between patients undergoing thyroidectomy with drains and those without drains, shown overall and stratified by study design. Squares represent study-specific mean differences, with square size proportional to study weight; horizontal lines indicate 95% confidence intervals; diamonds represent pooled effect estimates with 95% confidence intervals; the vertical dotted line indicates the line of no effect [21,22,23,24,25,26,27,28,29,30,31,34,38,39,40,42,46,47,48].

Postoperative length of hospital stay was reported in 18 studies including 2279 patients, of whom 1212 were managed with drains and 1067 without drains. The pooled random-effects analysis demonstrated a significantly longer hospital stay in patients managed with drains compared with those without drains, with a pooled mean difference of 1.96 days (95% CI 0.42–3.50; *p* = 0.016) (Figure 7).

### 3.3. Sensitivity and Robustness Analyses

Sensitivity analyses confirmed the robustness of the primary findings. Leave-one-out analyses demonstrated that the pooled estimates for all binary outcomes were not driven by any single study (Figures S1, S8, S12–S14). For SSI and hematoma, influence diagnostics and Baujat plots did not identify studies exerting a disproportionate influence on the overall effect estimates (SSI: Figures S2 and S3; hematoma: Figures S9 and S10).

For SSI, additional sensitivity analyses—including the exclusion of double-zero studies, the application of a Peto fixed-effect model, one-stage generalized linear mixed-effects modelling, and stratification by study size—yielded results consistent with the main analysis (Figures S4–S7). Similarly, the exclusion of double-zero studies for hematoma, when applicable, did not materially affect the pooled estimate (Figure S11).

For length of hospital stay, leave-one-out and influence diagnostics supported the stability of the pooled effect (Figures S15–S17). Meta-regression analyses exploring the association of length of stay with the year of publication, study design, and total sample size are presented in Figures S18–S20.

## 4. Discussion

The current study assessed the association between postoperative cervical drainage and early postoperative outcomes following thyroidectomy. According to the findings, drain use was associated with higher odds of surgical site infections and a longer postoperative length of hospital stay, whereas no statistically significant associations were observed for cervical hematoma, seroma formation, postoperative bleeding, or reoperation.

These findings should be interpreted within the context of the historical rationale for drainage, the evolution of thyroid surgical practice, and the structure of the currently available evidence.

### 4.1. Conceptual Framework of Cervical Drainage in Thyroidectomy

Routine cervical drainage after thyroidectomy was historically adopted as a preventive measure against postoperative hemorrhage and cervical hematoma, complications that may lead to acute airway compromise despite their low incidence [51,52]. The practice was grounded in anatomically plausible concerns and became established during periods characterized by less standardized hemostatic techniques, limited perioperative monitoring, and routine inpatient postoperative care [2,53]. Implicit in this approach were two key assumptions: first, that drains could prevent clinically relevant fluid accumulation or bleeding and second, that drain output could serve as an early indicator of postoperative hemorrhage [5,54].

From a pathophysiological and technical perspective, increasingly debated in contemporary thyroid surgery, surgical drains primarily evacuate superficial fluid collections and may not reliably decompress rapidly expanding deep bleeding within the thyroid bed, which is considered one of the mechanisms underlying clinically significant cervical hematoma [55,56,57]. Moreover, the obstruction of drains by clotted blood is a well-recognized phenomenon, potentially limiting the reliability of drain output as an early warning indicator, particularly in rapidly evolving bleeding events [4,58]. Consequently, the prevention and early detection of postoperative hemorrhage depends predominantly on meticulous intraoperative hemostasis, appropriate perioperative blood pressure control, and structured postoperative surveillance, whereas timely clinical recognition—rather than drain output—remains the critical determinant of patient safety [6,57,59].

The relevance of routine cervical drainage must also be interpreted in the context of the substantial evolution of thyroid surgical practice. Advances in operative technique, including refined dissection methods and the widespread adoption of energy-based vessel sealing devices, are associated with improved hemostatic control and persistently low rates of major bleeding complications in thyroidectomy series [60,61]. In parallel, large observational cohorts and multicentric studies have suggested that the risk of postoperative hematoma is primarily determined by patient- and procedure-specific factors—such as the extent of surgery, comorbidities, and perioperative management—rather than by routine perioperative interventions such as drain placement [3,62,63].

### 4.2. Outcome-Specific Interpretation of Early Postoperative Endpoints

In light of these considerations, the contemporary debate surrounding cervical drainage has shifted from routine application toward a more outcome-specific evaluation of its clinical impact. This necessitates separate examination of bleeding-related events, wound morbidity, and recovery-related parameters.

#### 4.2.1. Cervical Hematoma and Postoperative Bleeding

No statistically significant association was observed between cervical drainage and either cervical hematoma or postoperative bleeding in the present meta-analysis. When analyzed as distinct bleeding-related endpoints, routine drain placement did not demonstrate a protective effect.

These results mirror earlier investigations reporting no reduction in postoperative hematoma or hemorrhage among drained patients [36,54,55]. Larger comparative analyses have similarly failed to identify a protective association between routine drainage and bleeding-related outcomes [64,65,66]. Population-level data further demonstrate that clinically significant postoperative hematoma occurs at low baseline rates in contemporary thyroidectomy practice [57,63,67].

#### 4.2.2. Surgical Site Infections and Seroma Formation

A different pattern emerged for wound-related outcomes. Drain use was associated with a statistically significant increase in the odds of surgical site infection, whereas no significant association was observed for seroma formation.

The elevated infection risk aligns with prior systematic reviews documenting higher rates of wound-related complications among drained patients [68,69,70]. Existing literature has repeatedly emphasized drain-related wound morbidity in the absence of demonstrable benefit in preventing clinically relevant postoperative fluid accumulation [66,68].

Regarding seroma formation, comparative evidence has not consistently shown a reduction in clinically meaningful fluid collections with drain use [69,70]. Moreover, variability in the definition and ascertainment of postoperative seroma across studies has been recognized as a methodological constraint that may obscure true between-group differences [68,69].

#### 4.2.3. Length of Hospital Stay

Beyond complication rates, recovery metrics were also affected. Cervical drainage was associated with a significantly longer postoperative length of hospital stay compared with no-drain management.

In published analyses, extended length of stay has frequently been described alongside wound-related complications and delayed discharge pathways, without parallel reductions in clinically significant postoperative events [68,69,70].

Recovery-related outcomes remain inconsistently reported across thyroidectomy studies; nevertheless, contemporary syntheses increasingly emphasize the importance of avoiding perioperative practices that may extend hospitalization in the absence of measurable safety benefit [66,68].

### 4.3. Clinical Complexity and Contextual Considerations

Although the present analysis does not support routine cervical drainage after thyroidectomy, interpretation of the findings should consider that certain operative contexts are not uniformly represented in the available comparative literature.

More extensive procedures, including total thyroidectomy combined with neck dissection, have been associated with higher rates of postoperative hematoma in large population-level analyses [57,63]. However, stratified reporting according to surgical extent was inconsistent across the included studies, limiting the evaluation of whether drainage effects differ in such settings. Similarly, reoperative thyroid surgery has been linked to increased technical complexity and complication risk compared with primary procedures [3]. This subgroup was not systematically analyzed in most comparative datasets.

Patient-level risk modifiers, including poorly controlled hypertension and anticoagulant exposure, have also been associated with postoperative bleeding in observational cohorts [63]. These variables were variably reported and could not be incorporated into subgroup analyses within the current meta-analytic framework.

Furthermore, contemporary multidisciplinary guidance emphasizes that early clinical recognition and rapid intervention remain central to the management of post-thyroidectomy hemorrhage, without endorsing routine drainage as a preventive strategy [6]. Institutional differences in postoperative monitoring, discharge protocols, and access to immediate airway management were not consistently described in the included studies and therefore could not be evaluated as contextual modifiers of drainage practice. These considerations do not establish a protective role for drainage in specific subgroups but rather underscore the limited granularity of currently available comparative evidence in complex or high-risk operative scenarios.

### 4.4. Strengths and Limitations

#### 4.4.1. Strengths

The present meta-analysis examines postoperative outcomes associated with cervical drainage after thyroidectomy by integrating randomized controlled trials and non-randomized comparative studies within a single analytical framework. This approach reflects the structure of the available evidence in thyroidectomy and permits the evaluation of outcomes derived from both trial-based settings and routine clinical practice, where randomized data remain limited for several postoperative endpoints.

A key strength of this study lies in its outcome-specific and clinically structured design. Early postoperative events were analyzed as distinct endpoints, with explicit separation of cervical hematoma, postoperative bleeding, reoperation, seroma formation, surgical site infection, and length of hospital stay. Outcomes that are frequently grouped together in earlier syntheses—particularly bleeding-related complications—were examined independently, acknowledging their differing clinical implications and management pathways.

Methodological rigor represents an additional strength. The analytical strategy was prespecified to address challenges inherent to thyroidectomy research, including low event rates for serious complications and the selective nature of drain placement. Methods appropriate for sparse-event data were applied, and pooled estimates were interpreted conservatively, with attention to potential confounding by indication. Emphasis was placed on the consistency of effect direction and robustness across sensitivity analyses rather than on causal inference based on statistical significance alone.

The analysis also incorporates contemporary surgical context. Advances in operative technique, perioperative hemostatic control, and structured postoperative monitoring have substantially altered the baseline risk profile of modern thyroidectomy. Accordingly, the synthesis focuses on conventional cervical thyroidectomy, as endoscopic and robotic remote-access techniques remain variably adopted and unevenly distributed across clinical settings and combining such approaches without stratification would limit the interpretability and generalizability of pooled estimates to routine clinical practice [71,72,73].

The simultaneous evaluation of safety-related and recovery-related outcomes within a unified analytical framework constitutes a further strength. This integrative perspective situates cervical drainage within broader considerations of perioperative safety, postoperative recovery, and resource utilization, facilitating a balanced clinical interpretation of the observed associations.

#### 4.4.2. Limitations

Several limitations of the present analysis warrant consideration. First, drain placement in thyroidectomy is rarely random in routine clinical practice. The decision to insert a drain is frequently influenced by surgical complexity, disease extent, intraoperative findings, and institutional practice patterns. Consequently, drainage may act as a surrogate marker of operative difficulty or baseline risk rather than an isolated intervention. Although adjusted and pooled analyses were performed, residual confounding related to case-mix differences cannot be excluded at the aggregate level.

Second, clinically relevant bleeding-related events—including cervical hematoma, postoperative hemorrhage, and reoperation—occur infrequently in contemporary thyroidectomy. Despite the inclusion of a large cumulative sample, low event rates inherently limit statistical precision and may result in wide confidence intervals for some endpoints. Consequently, the absence of statistically significant associations for some outcomes should not be interpreted as definitive evidence of equivalence. Confidence intervals should be considered when assessing the range of plausible effect sizes, as imprecision remains possible despite pooled analysis.

Third, heterogeneity in outcome definitions and reporting across studies restricts direct comparability. Bleeding-related endpoints were variably defined, ranging from return to theatre to clinically or radiologically detected hematomas. Similar variability was observed in the definition and ascertainment of seroma formation and wound-related complications, potentially attenuating true between-group differences.

Fourth, the geographical distribution of included studies limits external validity. A substantial proportion of available data derives from Asian cohorts, with comparatively limited representation from other populations. Differences in perioperative pathways, discharge protocols, and institutional standards may therefore influence generalizability.

Fifth, technical aspects of drainage were inconsistently reported and could not be evaluated. These include the precise placement of the drain (within the thyroidectomy incision or through a separate stab incision), drain characteristics, and specific operative contexts such as drainage following sternotomy for ectopic mediastinal thyroid. The absence of such procedural detail limits more granular interpretation.

Finally, patient-centered outcomes—including postoperative pain, drain-related discomfort, quality of life, and satisfaction—were inconsistently documented and could not be synthesized quantitatively. This restricts comprehensive evaluation of the broader clinical impact of drainage beyond conventional morbidity endpoints.

## 5. Conclusions

This systematic review and meta-analysis evaluated the association between postoperative cervical drainage and postoperative outcomes following thyroidectomy, integrating evidence from both randomized controlled trials and non-randomized comparative studies. Across the analyzed outcomes, routine drain use was associated with adverse recovery-related outcomes, most notably higher odds of surgical site infection and prolonged postoperative length of hospital stay.

These findings suggest that, within the context of thyroidectomy, routine cervical drainage does not confer a measurable protective effect on clinically meaningful postoperative complications. Advances in operative technique, together with structured perioperative hemostatic strategies and enhanced postoperative monitoring, have substantially reduced the baseline bleeding risk following thyroidectomy. Within this modern surgical environment, the traditional rationale for routine drainage appears increasingly unsupported by outcome-based evidence.

Importantly, the results should not be interpreted as evidence against selective drain use in all clinical circumstances. The available literature remains limited by low event rates for major complications, variability in outcome definitions, and the discretionary nature of drain placement in routine practice. In specific clinical contexts characterized by greater operative extent, reoperative procedures, or elevated bleeding risk, the current evidence base does not permit definitive conclusions regarding the potential differential impact of drainage. Findings should be interpreted as associations rather than proof of causality; robustness was assessed through study-design-stratified presentation and prespecified RCT-only sensitivity analyses. Residual confounding and differences in outcome ascertainment or surveillance practices across studies may have influenced the observed effect estimates.

The present findings further underscore the need for future research focused on standardized outcome reporting, contemporary surgical cohorts, and high-risk subgroups that are underrepresented in existing literature. Studies incorporating modern surgical techniques, including minimally invasive, endoscopic, and robotic approaches, as well as enhanced recovery and outpatient care pathways, are needed to clarify whether drainage practices should differ across evolving operative contexts.

Until such evidence becomes available, the routine use of cervical drainage after thyroidectomy cannot be supported based on current data. Decisions regarding drain placement should instead be individualized, guided by operative findings, patient-specific risk factors, and the availability of structured postoperative monitoring, rather than applied as a default component of thyroid surgical care.

## Figures and Tables

**Table 1:** The main characteristics of the included studies.

First Author	Year	Continent	Study Design	Sample Size (*n*)			Males, %	Mean Age (Years)
				Total	Drain Group	No-Drain Group		
Abaszadeh [21]	2017	Asia	Randomized controlled trial	180	90	90	0.21	41.44
Al-Habsi [22]	2016	Asia	Retrospective case–control	250	202	48	NA	40.80
Al-Qahtani [23]	2017	Asia	Randomized controlled trial	108	54	54	0.13	38.02
Alamani [24]	2014	Asia	Randomized controlled trial	66	32	34	0.18	44.25
Arif [25]	2022	Asia	Randomized controlled trial	60	30	30	0.52	NA
Bachani [26]	2020	Asia	Prospective comparative	98	49	49	0.09	NA
Chalya [27]	2011	Africa	Randomized controlled trial	62	32	30	0.07	48.9
Chen [28]	2022	Asia	Retrospective case–control	110	55	55	0	NA
Colak [29]	2008	Asia	Randomized controlled trial	116	58	58	0.21	NA
Deveci [30]	2013	Asia	Randomized controlled trial	400	200	200	0.12	46.80
Dogar [31]	2019	Asia	Randomized controlled trial	62	31	31	0.23	43.05
George [32]	2023	Asia	Randomized controlled trial	54	27	27	0.06	44.80
Hau [33]	2023	Asia	Randomized controlled trial	84	42	42	0.07	47.12
Hussain [34]	2019	Asia	Randomized controlled trial	200	100	100	0.24	45.60
Ishaq [35]	2008	Asia	Randomized controlled trial	60	30	30	0.07	39.20
Khanna [36]	2005	Asia	Randomized controlled trial	102	51	51	0.13	34.60
Lee [37]	2006	Asia	Randomized controlled trial	198	101	97	0.12	47.60
Memon [38]	2012	Asia	Randomized controlled trial	60	30	30	0.10	31.75
Mohialdeen [39]	2016	Asia	Retrospective cohort	102	48	54	0.26	43.00
Muthaa [40]	2013	Africa	Randomized controlled trial	90	45	45	0.11	42.60
Naeem [41]	2022	Asia	Randomized controlled trial	40	20	20	0.38	44.45
Nawaz [42]	2015	Asia	Randomized controlled trial	68	32	36	NA	42.00
Neary [43]	2012	Europe	Randomized controlled trial	93	49	44	0.16	51.30
Rahim [44]	2021	Asia	Randomized controlled trial	30	15	15	0	36.07
Saleem [45]	2023	Asia	Randomized controlled trial	108	54	54	0.39	NA
Kalemera Ssenyondo [46]	2013	Africa	Randomized controlled trial	68	34	34	0.09	46.00
Suslu [47]	2006	Asia	Randomized controlled trial	135	68	67	0.19	NA
Wang [48]	2023	Asia	Randomized controlled trial	104	52	52	0.12	35.70
Wijesinghe [49]	2016	Asia	Randomized controlled trial	50	25	25	0.14	43.30
Yasin [50]	2014	Asia	Randomized controlled trial	40	20	20	0.20	NA

**Table 2:** Pooled effect estimates for postoperative outcomes comparing drain versus no-drain thyroidectomy.

Outcome	Studies	Pooled Effect Estimate			Patients Contributing to the Analysis (n)		
		OR/MD ^1^	95% CI ^2^	*p*-Value	Drain	No-Drain	Total
Cervical hematoma	26	1.28	0.93–1.75	0.124	1568	1414	2982
Surgical site infection	27	2.04	1.46–2.85	0.0002	1518	1368	2886
Seroma	26	0.95	0.70–1.30	0.750	1548	1397	2945
Postoperative bleeding	15	1.26	0.85–1.86	0.228	1063	900	1963
Reoperation	15	0.89	0.59–1.32	0.525	878	878	1756
Length of hospital stay (days)	18	1.96	0.42–3.50	0.016	1212	1067	2279

^1^ OR: odds ratio; MD: mean difference. ^2^ CI: Confidence interval. Random-effects models were applied for all pooled analyses. Positive OR or MD values indicate higher odds or longer hospital stay in the drain group.

## Data Availability

All data analyzed in this study are derived from previously published articles included in the systematic review. Extracted and synthesized data supporting the findings of this study are available from the corresponding author upon reasonable request.

## Supplementary Materials

The following supporting information can be downloaded at: https://doi.org/10.5281/zenodo.19192893. Table S1. PRISMA 2020 reporting checklist; Table S2. Detailed search strategies by database; Table S3. Risk of bias assessment of included studies; Figure S1: Leave-one-out analysis for surgical site infection (SSI); Figure S2: Influence diagnostics for surgical site infection (SSI); Figure S3: Baujat plot for surgical site infection (SSI); Figure S4: Sensitivity analysis for SSI excluding studies with zero events in both arms; Figure S5: Sensitivity analysis for SSI using a Peto fixed-effect model; Figure S6: One-stage generalized linear mixed-effects model (GLMM) for SSI; Figure S7: Subgroup analysis of SSI stratified by study size; Figure S8: Leave-one-out analysis for hematoma; Figure S9: Influence diagnostics for hematoma; Figure S10: Baujat plot for hematoma; Figure S11: Sensitivity analysis for hematoma excluding studies with zero events in both arms; Figure S12: Leave-one-out analysis for seroma; Figure S13: Leave-one-out analysis for postoperative bleeding; Figure S14: Leave-one-out analysis for reoperation; Figure S15: Leave-one-out analysis for length of hospital stay; Figure S16: Influence diagnostics for length of hospital stay; Figure S17: Baujat plot for length of hospital stay; Figure S18: Meta-regression of length of hospital stay by year of publication; Figure S19: Meta-regression of length of hospital stay by study design; Figure S20: Meta-regression of length of hospital stay by total sample size.
